# Correction: Progressive decline in hippocampal CA1 volume in individuals at ultra-high-risk for psychosis who do not remit: findings from the longitudinal youth at risk study

**DOI:** 10.1038/s41386-019-0477-6

**Published:** 2019-08-14

**Authors:** New Fei Ho, Daphne J. Holt, Mike Cheung, Juan Eugenio Iglesias, Alex Goh, Mingyuan Wang, Joseph K. W. Lim, Joshua de Souza, Joann S. Poh, Yuen Mei See, R. Alison Adcock, Stephen J. Wood, Michael W. L. Chee, Jimmy Lee, Juan Zhou

**Affiliations:** 1 Institute of Mental Health, Singapore, Singapore; 2 Neuroscience and Behavioral Disorders Program, Center for Cognitive Neuroscience, Duke-National University of Singapore Graduate Medical School, Singapore, Singapore; 3 Department of Psychiatry, Massachusetts General Hospital and Harvard Medical School, Boston, MA USA; 4 Department of Psychology, National University of Singapore, Singapore, Singapore; 5 University College London, London, UK; 6 Center for Cognitive Neuroscience, Duke University, Durham, NC USA; 7 School of Psychology, University of Birmingham, Birmingham, UK

**Correction to:** *Neuropsychopharmacology* 10.1038/npp.2017.5, published online 12 January 2017

The original version of this Article contained an error in the spelling of the author R. Alison Adcock, which was incorrectly given as Alison R. Adcock.

